# EtG Quantification in Hair and Different Reference Cut-Offs in Relation to Various Pathologies: A Scoping Review

**DOI:** 10.3390/toxics10110682

**Published:** 2022-11-11

**Authors:** Valentina Triolo, Mario Spanò, Roberto Buscemi, Simona Gioè, Ginevra Malta, Marija Čaplinskiene, Fabio Vaiano, Elisabetta Bertol, Stefania Zerbo, Giuseppe Davide Albano, Antonina Argo

**Affiliations:** 1 Policlinic Hospital, AOUP “P. Giaccone”, Via del Vespro 129, 90127 Palermo, Italy; 2 Section of Legal Medicine, Department of Health Promotion, Mother and Child Care, Internal Medicine and Medical Specialties, University of Palermo, 90129 Palermo, Italy; 3 State Forensic Medicine Service, Mykolas Romeris University, Ateities St. 20, LT-08303 Vilnius, Lithuania; 4 Forensic Toxicology Division, Department of Health Sciences, University of Firenze, 50134 Firenze, Italy

**Keywords:** forensic medicine, ethanol, alcohol, ethyl glucuronide (EtG), hair, diabetes mellitus, liver disease, renal failure, forensic toxicology

## Abstract

Ethyl glucuronide (EtG) is a non-volatile, non-oxidative, hydrophilic, and stable ethanol phase II metabolite. EtG is produced through ethanol glucuronidation by UDP-glucuronosyltransferase (UGT), a phase II enzyme. EtG can be extracted from different biological matrices, including keratin ones, such as hair or nails. The purpose of this scoping review is to describe the relationship between EtG levels in hair and some of the most common and frequent pathological conditions and verify whether different reference cut-offs in relation to various pathologies have been identified in the scientific literature. In fact, in-depth knowledge of the influence of pathologies, such as diabetes mellitus, hepatic and renal dysfunction, on EtG production and its storage in keratin matrices would allow a more appropriate interpretation of obtained data and rule out false positives or false negatives. This scoping review is based on bibliographic research carried out on PubMed regarding the quantification of EtG in hair of subjects affected by different pathological conditions. According to the scientific literature, the main and most common pathologies that can affect the concentration of EtG in hair are liver and kidney diseases and diabetes. The EtG quantification analytical data should be interpreted carefully as they may have a great impact in both forensic and clinical contexts.

## 1. Introduction

The detection and quantification of chronic alcohol consumption is important for forensic and clinical purposes as it affects any legal actions, as to interfere with imputability of suspect, and/or assistance decisions concerning a subject suspected of chronic alcohol intake. It has implications in the workplace, for example, with the possibility of suspension of driver’s or pilot’s license, in childcare activities, or in care settings, such as adding or withdrawing from liver transplant waiting lists, for example. Furthermore, from a clinical point of view, objective monitoring of alcohol withdrawal in alcohol-dependent patients may affect the assessment of a treatment’s effect [1]. Markers are needed to identify and quantify alcohol consumption in order to monitor abstinence/consumption. These markers should differ between “at-risk” drinkers, “no-risk” drinkers and subjects in withdrawal phase; and detect alcohol consumption/abstinence over a long period of time (i.e., after complete alcohol elimination from the body) [2].

Direct and indirect markers are available. Some of them, however, despite being widely available and relatively inexpensive, show low specificity and sensitivity for detecting alcohol consumption. For this reason, some authors have proposed combining them to allow greater specificity. Since the 2000s, EtG has been identified as an excellent direct-type reference marker. EtG is a non-volatile, non-oxidative, hydrophilic, and stable phase II ethanol metabolite, with a molar weight of 222 g/mol. This metabolite is produced through the glucuronidation of ethanol by UDP-glucuronosyltransferase (UGT), which adds a glucuronic acid moiety to the ethanol molecule. EtG can be extracted from different biological matrices: blood and urine (which only allow alcohol detection in a short period), or keratin matrices such as nails and hair. The toxicological analysis of these matrices allows verification of the presence of toxic agents and drugs over a period of months; thus, the window of time detection of substances is usefully extended. Therefore, in addition to a good sensitivity and specificity, a great advantage of this marker is that it detects chronic alcohol intake over a long period of time, which can range from months to years, depending on sample length, through its quantification in keratin matrices (mostly hair).

According to the most recent review by the Society of Hair Testing (SoHT) [3], an EtG concentration ≤ 5 pg/mg in the proximal segment of the scalp hair with a length of 3 cm up to 6 cm does not contradict self-reported abstinence, while a concentration > 5 pg/mg strongly suggests repeated alcohol consumption, whereas a concentration ≥ 30 pg/mg strongly suggests chronic excessive alcohol consumption. Thus, based on internationally adopted cut-offs, abstinence and chronic excessive alcohol consumption can be detected. A hair EtG concentration between 5 and 30 pg/mg is considered as a strong indicator of regular alcohol consumption. Unfortunately, direct and indirect markers, including EtG, can undergo changes due to several factors, including intrinsic factors, for example, sex, age, comorbid conditions that increase enzyme levels but that are not related to alcohol consumption per se; and extrinsic factors, such as cosmetic treatment and/or hair hygiene.

The concentration of EtG in hair depends on exposure time and EtG concentration in the blood; in turn, the concentration of EtG in blood is strongly dependent on individual metabolism. Therefore, all pathological changes that affect ethanol metabolism should be considered potential confounders for hair EtG concentration and, consequently, the final data, used both for forensic and clinical purpose, should be interpreted with great caution. According to the scientific literature, the main and most frequent pathologies that can influence the concentration of EtG in hair are hepatic and kidney diseases and also diabetes. For example, it was suggested by Crunelle et al. that the alteration of UDP-glucuronosyltransferase (UGTs), typical of some liver disease such as Gilbert’s syndrome, causes lower EtG levels [1]. The purpose of this scoping review is to describe, through literature search research, the main and most frequent pathological conditions that may affect EtG levels in hair and to verify if different reference cut-offs in relation to various pathologies have been identified in the scientific literature. In fact, in-depth knowledge of the influence of these pathologies in the production of EtG and its storage in keratin matrices would allow a more appropriate interpretation of analytical data and rule out false positives or false negatives, especially reported cases of abstinence.

## 2. Materials and Methods

The present scoping review is based on data collected using a PubMed search on the quantification of EtG in the hair of subjects affected by different pathological conditions from 1970 to 2022. The search was performed using the terms “liver cirrhosis”, “liver disease”, “hepatitis”, “Gilbert syndrome”, “diabetes”, “renal failure” and the terms (or their combination) “hair”, “ethyl glucuronide”, “EtG”, “alcohol”, “marker”, “endogenous ethanol”, “auto-brewing”, “abstinence”, “alcohol abuse” and “Society of Hair Testing”. This was further supplemented by a Google Scholar search, by using the relevant items of research.

## 3. State of the Art of EtG Chemical Analysis and Forensic Significance

After ethyl alcohol intake, biochemical changes occur and can be evaluated by analytical laboratory techniques [4,5]. Markers of alcohol consumption can be classified as non-specific markers, as they do not provide direct information on quantitative alcohol consumption, such as liver enzymes (GGT, AST, ALT); and specific markers, which in turn provide a quantitative measure of metabolized ethanol, such as carbohydrate-deficient transferrin (CDT) and ethyl glucuronide (EtG) [6]. CDT is a specific but indirect marker because it gives a measure of changes caused by ethanol in transferrin composition; it is determined both by HPLC and capillary electrophoresis. Ethyl glucuronide is a direct non-oxidative polar and hydrophilic metabolite of ethanol, and its measurement provides a very reliable quantification of alcohol consumption [7]. It can be eliminated through both urine and sweat, but it can also be found in the hair; the concentration of EtG in hair is directly related to alcohol consumption. According to the Society of Hair Testing (SoHt), the EtG molecule is stable in the keratin matrix up to 12 cm. By dividing the hair into segments not less than 3 cm, we can examine the consumption of alcohol over time [3,8]. Some studies have shown a better reliability of the sample for hair length not less than 3 cm. The part closest to the skin provides information about recent consumption; the part furthest from the skin is useful for detecting ethanol intakes further back in time [9]. Considering that hair grows about 1 cm a month, the keratin matrix offers a time window of a few months. Dyes and bleaches can cause false negatives when measuring ethyl glucuronide; the use of alcoholic lacquers or lotions on the contrary can produce false positive results. Worthy of considering, some authors, however, have not found alterations in the presence of use of alcoholic lotions [10].

A typical procedure involves weighing 15 to 200 mg of hair [11]. Subsequently, the weighed amount is washed to eliminate fat and environmental contaminants of different nature that could lead to false results [12,13]. In the SoHT, washing with water prior to extraction is recommended. After this washing, hair may be dried with acetone or dichloromethan. Dichloromethane is often used, followed by methyl alcohol immediately after. The sample is dried at room temperature, and then the hair must be shredded with clean scissors to obtain segments of about 0.1–0.3 cm in length or it can be better crushed by a ball mill [14,15]. Extraction is usually performed with distilled water through simple incubation in an ultrasonic bath, or a combination of both methods. Distilled water proved to be a better solvent than methanol or a water/methanol mixture. Deuterated ethylglucuronide (d5-Etg) is used as an internal standard [16,17]. Sensitivity can be increased by one order of magnitude thanks to solid phase extraction. For derivatization, it is preferable to use pentafluoropropionic anhydride (PFPA) instead of heptafluorobutyric anhydride (HFBA) or bis(trimethylsilyl)trifluoroacetamide (BFA) 1% TMC. However, for GC-MS/MS, HFBA was revealed to be the optimal derivatization agent. After heating the sample for 30 min with the derivatizing agent at 60 °C, it is dried with nitrogen and the residue taken up with 50 microliters of ethyl acetate [18]. Finally, after plotting a calibration curve, 2 µL of sample solution are injected into GC-M system for quantification of the EtG in the sample being analyzed [19,20]. The gold standard is actually GC/MS/MS NCI or LC-MS/MS.

## 4. Renal Dysfunction

### 4.1. Premise

EtG levels in hair (hEtG) are used as an indicator of long-term alcohol consumption and the cut off of 30 pg/mg in the proximal 3 cm hair segment has been suggested as a limit value for identifying a high daily intake of alcohol (above 60 g/day) [21] in the last three months [22]. In patients awaiting liver transplantation, the hEtG determination is widely used as it represents a useful direct marker that provides information on alcohol intake in the period prior to 3–6 months to the execution of the determination. However, a high percentage of patients with advanced liver cirrhosis or awaiting liver transplantation have impaired renal function. Therefore, it is particularly important from a clinical point of view to understand the impact of renal dysfunction and hemodialysis on hEtG outcomes.

Several studies showed significantly higher levels of hEtG in subjects with impaired renal function compared to healthy individuals. In addition, in the patient group, a significant correlation was found between hEtG levels and estimated glomerular filtration rate (eGFR) and serum creatinine levels [23]. Some studies have also shown prolonged urinary detection times for EtG and ethyl sulfate (EtS) in patients with impaired renal function [24]. Since renal excretion is the main route of EtG and EtS elimination, in case of alterations in renal function, after taking an identical amount of alcohol, higher blood concentrations of EtG and EtS could be detected compared to healthy population. Higher EtG blood levels could result in greater incorporation into hair and, as a result, higher levels of EtG in this matrix.

### 4.2. Discussion

Some studies [25] have revealed that hEtG determination is a useful withdrawal marker in patients with compensated renal function, with a specificity of 92%. Specificity decreased in patients with moderate and severe renal dysfunction, but in this group the possibility of identification of abstainers is between 87% and 82% by verifying a negative hEtG result. In addition, a negative hEtG result can exclude regular consumption of more than 10 g/day in patients with advanced or moderate renal dysfunction (negative predictive value, NPV 100%), and in almost all patients with compensated or moderately reduced renal function (NPV 87%). On the other hand, a positive hEtG test can correctly identify any alcohol consumption only in 71–78% of cases, and positive predictive value (PPV) is not significantly different between subjects with and without renal dysfunction, but in these two groups hEtG concentration is much higher in patients with renal dysfunction in relation to the amount of alcohol consumed. Furthermore, in relation to the degree of renal dysfunction, (compensated, moderately reduced, or severe), the hEtG concentration per gram of alcohol consumed per day for a period of 3 months (hEtG/EDI, daily intake of ethanol) was significantly higher in patients with severe renal dysfunction. After consuming a low amount of alcohol (mean EDI: 3 g/day), patients with glomerulus filtration rate (GFR) < 30 mL/min showed a very high average hEtG concentration of 145 pg/mg, while the average consumption of 17 g/day resulted in a mean hEtG concentration of 52 pg/mg and an average consumption of 16 g/day resulted in a mean hEtG concentration of 74 pg/mg in patients with moderate and absent renal impairment, respectively. In addition, in almost all patients (86%) with advanced renal dysfunction, hEtG concentrations were above 30 pg/mg, although none of these subjects reported consumption of more than 10 g of ethanol per day. Considering the standardized cut-offs, this value could have erroneously indicated alcohol abuse in patients with advanced renal failure.

In Mosebach et al.’s study, most patients with advanced renal dysfunction (15/18, 83.3%) were on hemodialysis [25]. In 71% (*n* = 5) of dialysis patients with any declared amount of alcohol intake, hEtG concentrations were very high (>30 pg/mg) despite a low daily intake of ethanol (<10 g/day). In addition, the hEtG concentration per gram of alcohol consumed per day was significantly higher in patients on dialysis (hEtG/EDI: 51.6) than in patients with moderate renal dysfunction (hEtG/EDI: 3.1; *p* = 0.016) or patients with GFR > 60 mL/min (hEtG/EDI: 4.6, *p* = 0.004). In a further study [26], twelve patients (eight men and four women) were included. Mean age was 64 years (range, 42 to 84) and the mean body mass index was 26.2 kg/m^2^ (range, 18.9–29.4). All three criteria for reduced renal function were present in all patients. The mean serum creatinine value was 189 mmol/L (range, 130 to 670). The estimated mean GFR was 29.2 mL/min/1.73 m^2^ (range 8.0 to 54.0) and all subjects had a diagnosis of reduced renal function in their medical record. All subjects had ALT, AST and GGT in the normal range (50 U/L for ALT and AST, 60 U/L for GGT); hEtG levels ranging between less than LOD and 134 pg/mg; and EDIs ranging between 0.1 and 12 g were found. The two highest hEtG values were observed in patients with the lowest renal function. In patients with impaired renal function (*n* = 12), there was a significant correlation between hEtG level (adjusted for EDI) and GFR (Spearman’s ρ = 0.61, *p* = 0.036). The correlation between hEtG (EDI-adjusted) and serum creatinine was slightly weaker and not statistically significant (Spearman’s ρ = 0.53, *p* = 0.076).

These disproportionately high hEtG concentrations in patients with renal dysfunction can be explained pathophysiologically. A delay in renal excretion of EtG (caused by renal pathology) causes higher blood levels of metabolite, resulting in a greater deposition of EtG in the hair [26]. In this case, a 3 cm long hair segment may not reflect alcohol consumption in the last three months, but rather a longer period during which EtG accumulates in the hair. In this regard, however, there are no studies in the literature that have been able to ascertain this last thesis. In addition, the excretion of glucuronic acid [27,28,29] and, to a lesser extent, conjugated sulfates [30], as well as other drugs [31], have been reported to be reduced in patients with impaired renal function. It was observed that EtG and EtS had considerably longer blood half-lives in a patient with kidney disease [32]. Another study indicated that excretion of EtG and EtS is delayed in patients with kidney disease and the effect appears to be similar for both ethanol (EtOH) conjugates [24]. The kinetics and prolonged urinary detection times of EtG and EtS in this population deserve further studies. Studies in the literature, however, have limitations: the first determined by the fact that alcohol consumption is self-declared by the subject. Considering that alcohol consumption is an exclusion criterion for liver transplantation, some of the patients awaiting transplantation may have denied alcohol intake, although the questionnaires were provided anonymously, and patients were informed that the data would not be used for the decision process. However, it is true that false statements should be similarly distributed among different patient groups, so as not to presumably result in a significant difference in false positives between groups with different GFR. 

Moreover, since EDI declared by the patient is probably quite inaccurate and it is only a rough estimate, the calculated concentration of hEtG per gram of alcohol consumed (hEtG/EDI) can be taken as an approximate figure. It should be borne in mind that hEtG concentrations allow only a semi-quantitative estimation of the amount of alcohol consumed. In addition, the studies are limited by the number of subjects for statistical analysis. 

Finally, some of the patients whose hair was analyzed regularly used products such as hairspray, headbands, or tonics. It cannot be excluded that these products may have been contaminated with EtG and therefore have produced a false positive result. Previously, it has been reported that EtG may be present in commercially available hair care products [33,34]. In the international scientific literature, numerous studies document an increased risk of getting a false diagnosis of alcoholism among patients with kidney disease who socially drink [23,24,25,26]. In summary, the diagnostic accuracy of the hEtG test is worse in patients with advanced renal dysfunction than in patients without clinically relevant renal impairment. Therefore, the interpretation of EtG levels in hair among patients with reduced renal function should be performed with caution, particularly when evaluating the inclusion of patients on the transplant list. In patients with advanced kidney disease, a negative hEtG result may still rule out regular alcohol consumption above 10 g/day and may confirm alcohol withdrawal with a high probability of about 82%. However, a positive result does not exclude abstinence in the last three months with certainty. In addition, the hEtG concentration greatly overestimates the amount of alcohol consumed, in relation to the standardized parameters on studies performed with healthy patients. Particularly in patients on hemodialysis, hEtG monitoring appears to be very unreliable [25]. Considering the possible consequences of a false positive diagnosis of alcohol abuse based on hEtG in patients with kidney disease, further studies should be undertaken to investigate the relationship between alcohol consumption, the degree of renal failure and hEtG, as well as to evaluate new cut-off levels and detection periods.

## 5. Liver Dysfunction

### 5.1. Premise

After absorption, alcohol is metabolized in the liver—while a minor fraction is excreted unchanged in breath, sweat, and urine—primarily through oxidative pathways involving phase I enzymes such as alcohol dehydrogenase, aldehyde dehydrogenase, cytochrome CYP4502E1 and catalase, and secondarily through non-oxidative pathways involving phase II enzymes, such as glucuronidation catalyzed by UDP-glucuronosyltransferases (UGTs), which involve 0.2–0.6% of total ingested ethanol. UDP-glucuronosyltransferases (UGTs) are the primary phase II enzymes catalyzing the conjugation of glucuronic acid to the xenobiotics with polar groups for facilitating their clearance. These reactions occur: cofactor, enzyme, and the active substrate. In this way, starting from ethanol as active substrate, using UDP-glucuronic acid as a cofactor, UGTs enzymes catalyze the production of EtG. UGTs are a superfamily of enzyme that has several isoforms and any noted polymorphism in this family can potentially lead to a different expression of EtG production, as it could be theoretically affected by liver disease—and then it can theoretically affect the grade of internalization into keratin-based matrix. At the current state [33], SoHT states that a concentration ≤ 5 pg/mg EtG does not contradict self-reported abstinence, a concentration of ≥5 pg/mg strongly suggests repeated alcohol consumption, and a concentration ≥ 30 pg/mg strongly suggests chronic excessive alcohol consumption. 

### 5.2. In Vitro Studies on UGT Isoforms

Different in vitro and in vivo studies have been conducted to establish which UGT isoform is the main one involved in EtG formation, with not always consistent results. Foti and Fisher [35] incubated ethanol—concentrations of 0.1 and 5 mM—with a variety of recombinant UGT isoforms (UGT1A1, 1A3, 1A4, 1A6, 1A7, 1A8, 1A9, 1A10, 2B4, 2B7, 2B15, and 2B17) to determine the isoforms potentially responsible for catalyzing the addition of glucuronic acid to ethanol. They found that UGT1A1 and UGT2B7 were the two most prevalent isoforms involved in glucuronidation of ethanol. Al Saabi et al. [36] incubated 50 mM and 500 mM of ethanol with 12 recombinant UGT enzymes (UGT1A1, 1A3, 1A4, 1A6, 1A7, 1A8, 1A9, 1A10, 2B4, 2B7, 2B15, and 2B17) to identify UGTs involved in ethanol glucuronidation. They found that all tested isoforms, except UGT1A1, 1A6, and 1A10, can produce EtG in significant amounts and, among these, UGT2B7, followed by UTG1A9, exhibited the highest activity at both concentrations. Schwab and Skopp [37], in order to determine the UGT isoforms responsible for EtG production, incubated recombinant UGT ethanol concentrations of 50 mmol L^−1^ and 200 mmol L^−1^, finding that all UTGs under investigation (UGT1A1, 1A3, 1A4, 1A6, 1A9, 2B7, 2B10, 2B15) produced EtG in significant amounts. Among these UGTs, UGT1A9 and UGT2B7 had the highest activity for both ethanol concentrations, matching data obtained by Al Saabi et al. From the main in vitro studies carried out to find which isoform is the main isoform of UTG responsible for the ethanol glucuronidation, no univocal results emerged. The main isoforms appear to be UGT1A9 and UGT2B7, the latter being the only isoform to have the highest activity in all the studies considered. It is still uncertain about which UGT isoform is most involved in ethanol glucuronidation; though it seems that a variety of isoforms are involved in the process.

### 5.3. In Vivo Studies

#### 5.3.1. Gilbert’s Syndrome

Gilbert’s syndrome is a mild, usually asymptomatic, and relatively frequent (5% of the population) benign liver disease characterized by a partial deficit, between 20 and 70%, of the activity of the UGT1A1 isoform that causes an increase in bilirubin levels. Lower EtG production would be expected because of the UTG deficiency. Preliminarily it should be emphasized that our PubMed search did not show any results regarding the evaluation of EtG concentration in hair among patients with Gilbert’s syndrome. The only study that investigated the influence of Gilbert’s syndrome on EtG formation was conducted by Huppertz et al. on urine samples. Prior to this study, the influence of Gilbert’s syndrome on EtG production was only hypothesized [1]. Huppertz et al. [38] performed a drinking experiment in which 30 participants with Gilbert’s syndrome were asked to drink 0.1 L of sparkling wine (9 g of ethanol) within 5 min and then urine samples were collected to evaluate EtG formation. They found that the formation of EtG was not impaired in patients with Gilbert’s syndrome. This study contains several limitations, such as the low number of participants and the absence of a control group. However, it is possible that an impairment of UGT1A1, responsible for Gilbert’s syndrome, is masked by the combined activity of the other isoforms since in vitro studies showed that EtG formation is catalyzed by a variety of UGTs. Here, too, further studies are needed. Since Gilbert’s syndrome is relatively common, it is necessary to better understand if it causes lower EtG levels in the hair with the risk of generating false negatives.

#### 5.3.2. Liver Disease

Stewart et al. (2013) [39] evaluated the utility of hair EtG (hEtG) for detecting any drinking and moderate-to-heavy drinking in a cohort of 200 patients (107 men, 93 women) with liver disease in the past 90 days. Alcohol use was assessed by using alcohol timeline followback methods. Scalp hair was sampled and when hair length was greater than 3.8 cm, only the proximal 3.8 cm was utilized for EtG testing, while shorter lengths were processed in full. Hair EtG was assayed using a liquid chromatography-tandem mass spectrometry assay, with a limit of detection of 2 pg EtG/mg hair and a limit of quantitation of 8 pg/mg. Liver disease severity was estimated by the presence of cirrhosis vs. non-cirrhotic disease, with cirrhosis limited to subjects with biopsy-proven cirrhosis, current ascites from chronic liver disease or know esophageal varices. Nine patients were excluded from analysis based on exclusion criteria. Thus, 191 subjects were evaluated (53.4% men, 9.1% non-Hispanic whites, average age 51.2, 53.9% with cirrhosis, 57.1% reporting some drinking during the prior three months, 34.6% with detectable hEtG, and 27.2% with hEtG ≥ 30 pg/mg). Forty-six subjects reported some alcohol use (median 3.1 g per day) but did not have hEtG ≥ 8 pg/mg. Sixty-three subjects reported some alcohol use (median 56 g per day) and had hEtG ≥ 8 pg/mg. In the latter group, the median hair EtG concentration was 88 pg/mg. There was only modest correlation between average daily alcohol consumption during the past 90 days and hEtG concentration in subjects with detectable hEtG (Spearman correlation = 0.38, *p* = 0.002). They found that hEtG ≥ 8 pg/mg performed particularly well in detecting subjects averaging at least 28 g per day over the prior 3 months, with a sensitivity of 90% and specificity of 88%, while cut-off hEtG ≥ 30 pg/mg in detecting subjects averaging at least 28 g per day had 81% in sensitivity and 93% in specificity. In addition, logistic regression results demonstrated statistically significant interactions (*p* < 0.05) of drinking with gender and cirrhosis on the development of positive hair EtG results. Sensitivity and specificity estimates stratified by these factors suggested that detection of light drinking may be greater in men and detection of heavy drinking may be enhanced in cirrhotic subjects (detecting any drinking in cirrhotic patients in the past 90 days: sensitivity 65% and specificity 98%; while detecting cirrhotic patients averaging at least 28 g per day: sensitivity 100% and specificity 94%). The results of this study confirmed the utility of hEtG in patients with liver disease and that hEtG had a modest correlation with the total amount of alcohol ingested during the prior 3 months and that severity of liver disease may modestly influence the “accuracy” (sensitivity and specificity) of diagnosing alcohol consumption (i.e., somewhat better performance in cirrhotic vs. non-cirrhotic), but hEtG performed well regardless of cirrhosis. Limitations of this study were reliance on self-reported drinking to validate hEtG. The authors concluded that in their setting, hEtG is highly sensitive and specific and generally has a modest correlation with the total amount of alcohol ingested during the prior 90 days.

Sterneck et al. (2014) [40] conduced a prospective study to evaluate hEtG in a group of 63 orthotopic liver transplant candidates and after the authors compared hEtG analytical data with alcohol markers, urine EtG (uEtG), CDT, ethanol, and methanol, and then with patients’ self-reported history of alcohol consumption. Furthermore, authors included 25 patients with cirrhosis not because of alcohol, not presenting for liver transplantation and denying alcohol consumption in the preceding 180 days as a control group. Scalp hair samples were collected by cutting a 0.5 cm thick strand of hair near the skin. The proximal hair segment of 3 cm and, if available, the distal hair segments of 3 to 6 cm were analyzed for EtG. EtG concentration was determined with gas chromatograph 6890 and a mass selective detector 5975. The following cut-offs for hEtG were applied: <7 pg/mg: abstainers/rare drinking; 7–30 pg/mg: positive result (>10 g of ethanol per day); >30 pg/mg: positive result (>60 g of ethanol per day). hEtG was negative in all the hair samples collected in the control group, and one patient was positive for urinary EtG. Using the cut-off of 7 pg/mg for, sensibility, specificity, PPV and NPV were 76, 91, 71 and 94% respectively. The hEtG cut-off of 30 pg/mg increased diagnostic performance to 85, 97, 85 and 89%. The case of alcohol abstinence of only the prior 90 days was evaluated by the proximal hair segment using a cut-off of 30 pg/mg (sensitivity, 86; specificity, 98; PPV and NPV were 92 and 86%, respectively). In addition, authors, in order to investigate a potential impact of renal or liver dysfunction on hEtG concentrations, compared bilirubin, albumin, INR (International Normalized Ratio), Model for end-stage liver disease (MELD) and Child-Pugh score (used to assess the prognosis of chronic liver disease) at the time of sampling between transplant candidates with positive and negative hEtG and control patients, finding that there was no significant difference with respect to all tested parameters between the groups and no significant correlation between any of the parameters and hEtG values; however, they show no data. Streichert et al. (2015) [41] determined hEtG in a group of 104 liver transplant recipients, 31 (30%) with underlying alcoholic liver disease (ALD) and 73 (70%) with non-ALD. Patients were asked to complete a standardized written questionnaire to reveal their alcohol consumption in the previous 6 months. Scalp hair samples were collected by cutting a 0.5 cm thick strand of hair near the skin. The proximal hair segment of 3 cm and, if available, the distal hair segments of 3 to 6 cm were analyzed for EtG, reflecting alcohol consumption 0–3 and 3–6 months, respectively, prior to sampling. The following cut-offs for hEtG were applied: <7 pg/mg: negative result indicating abstaining or rare drinking; 7–30 pg/mg: positive result, which strongly suggests repeated consumption of alcohol (>10 g of ethanol per day); >30 pg/mg: high positive result indicating chronic excessive consumption (average consumption of ≥60 g of ethanol per day). Hair-ETG was determined by using liquid chromatography/tandem mass spectrometry. In this study, the detection of alcohol consumption was greatly improved among transplant recipients by implementing hEtG analysis. Compared to the determination of all serum and urinary markers of alcohol together (bilirubin, INR, mean corpuscular volume (MCV), GGT, ALT, AST), including uEtG, CDT, MeOH and EtOH, the determination of hEtG more than doubled the rate of detection of alcohol consumption from 7% to 17%. The determination of the concentration of EtG in the distal hair segments did not add much relevant information to the result of hEtG in the proximal segment. In patient self-reports, only 6% of all study participants stated regular alcohol consumption of a moderate or high amount in the previous 3 months, but as many as 14% of all transplant recipients had a positive hEtG result obtained from proximal hair segments. In addition, 43% and 3% of patients who stated only occasional or no alcohol consumption, respectively, had positive hEtG results. They concluded that hEtG determination is a valuable tool for revealing regular alcohol consumption among liver transplant recipients.

Verbeek et al. (2018) [42] tested the diagnostic accuracy of hEtG on a validation cohort of 101 subjects (43 heathy volunteers and 58 patients with alcoholic cirrhosis) and a clinical application group of 43 random patients with alcoholic cirrhosis (who denied chronic excessive alcohol use in the previous 90 days). Authors collected a self-reported questionnaire to assess alcohol intake, a blood sample and proximal 3 cm scalp hair strand to measure hEtG. O them, 29 patients (13 patients in the validation cohort and 16 in the clinical application group) underwent liver biopsy to assess the presence of hallmarks of ongoing excessive alcohol use. They found that among all 101 cirrhotic patients included in the study (43/43 of clinical application group and 58/101 of the validation cohort), total bilirubin, INR, MELD score, and creatinine did not differ between patients with low (<50 pg/mg; *n* = 67) and high (≥50 pg/mg; *n* = 34; for all *p* > 0.05) hEtG levels. There was no correlation between hEtG levels with MELD, bilirubin, or creatinine levels in the abstinent cirrhotic patients in the validation cohort (all *p* > 0.75) Cirrhotic patients with low hEtG levels (<50 pg/mg; *n* = 22) showed no hallmarks of ongoing excessive alcohol intake, except one abstinent patient. In the clinical application group, 35% of the patients with alcoholic cirrhosis (15/43; 5 of the 32 self-declared abstainers and 10 of the 11 self-declared moderate users) had hEtG ≥ 50 pg/mg, indicating chronic excessive alcohol use in the past 90 days. The sensitivity, specificity, PPV and NPV of hEtG in healthy volunteers were 47, 100, 100 and 19%, respectively, for detecting moderate alcohol intake (>0 g/day and <60 g/day corresponding to a hEtG concentration ≥7 pg/mg and <30 pg/mg) in the previous 3 months. In patients with alcoholic cirrhosis, hEtG demonstrated lower diagnostic accuracy in detecting moderate than excessive alcohol intake (≥60 g/day corresponding to hEtG ≥ 30 pg/mg): sensitivity, specificity, PPV and NPV to detect moderate alcohol consumption 67, 66, 38, 86, respectively; and sensitivity, specificity, PPV and NPV to detect excessive alcohol consumption 100, 97, 95, 100%. While using a cut-off of hEtG ≥ 50 pg/mg to identifying excessive alcohol consumption in patients with alcoholic cirrhosis, diagnostic accuracy increased to 100, 100, 100 and 100%. In addition, all the alcohol cirrhosis patients in whom chronic excessive alcohol consumption was excluded based on hEtG levels did not exhibit histological signs of such use. Authors concluded that applying a cut-off of hEtG ≥ 50 pg/mg results in hEtG better diagnostic accuracy for assessing chronic excessive alcohol use in the previous 90 days in patients with alcoholic cirrhosis, and since that hEtG < 50 pg/mg excludes chronic excessive alcohol consumption but does not allow distinction between abstinence or ongoing moderate alcohol intake in patients with alcoholic cirrhosis, hEtG levels < 50 pg/mg must be interpreted with caution.

The results are summarized in Table 1. In summary, from the evidence available to date, the scientific literature is solid regarding the usefulness of hEtG for detecting excessive alcohol consumption in patients with liver disease using a cut-off of hEtG ≥ 30 pg/mg, even if some authors suggested that a higher cut-off (i.e., ≥50 pg/mg) could improve diagnostic accuracy for detecting chronic excessive alcohol consumption in alcoholic cirrhosis patients. Great caution should be exercised when interpreting hEtG concentration between 5 and 30 pg/mg for detection of moderate or occasional alcohol intake in patients with liver disease, evaluating all pertinent case-related factors. In transplant setting, a false positive hEtG test result can incorrectly exclude a patient from transplantation since most transplant centers require a 6-month abstinence period as a primary condition to place the patient on the waiting list. However, hEtG is a marker capable of denying abstinence in most cases, and it has high PPV and specificity for detecting excessive alcohol intake, as it is an exclusive metabolite of ethanol. However, a higher cut-off for detecting moderate alcohol intake could increase false negative results, reducing test’s sensibility. Contrary to other biomarkers, data suggest that hEtG concentrations are not influenced by liver dysfunction, although EtG is produced by hepatocytes, but further studies are needed.

## 6. Diabetes Mellitus

### 6.1. Premise

In accordance with World Health Organization (WHO, Geneva, Switzerland), in recent decades the prevalence of diabetes mellitus has been steadily increasing. Above all, type 2 diabetes is growing, which represents about 90% of cases, strongly linked to excess weight, in turn referable to overeating and poor physical activity but also to the very structure of society. According to data provided by Italian National Institute of Statistics (ISTAT), in 2020 the prevalence of diagnosed diabetes was about 5.9% in Italy, equal to over 3.5 million people, with higher prevalence in male sex and with a slowly increasing trend in recent years. Furthermore, diabetes prevalence increases with increasing age up to 21% in people aged 75 or over [43], and, as can be seen from ISTAT data, it has almost doubled in thirty years (only 2.9% of population in 1980 was affected). Therefore, it is evident that in diabetic patients the evaluation of the variations in quantification of alcohol consumed assumes considerable importance in social and medico-legal fields [44].

### 6.2. Discussion

Examination of the available literature shows that various markers used in the assessment of excessive alcohol consumption are susceptible to alteration in diabetic patients, in particular quantification of CTD (carbohydrate-deficient transferrin) [45] and EtG. We observed that EtG levels can undergo in plus or minus variations in case of impaired metabolism, with potential false positive or false negative results, and hair EtG values may vary in relation to amount of ethanol in blood and urine. Unfortunately, it should be noted that, in particular for medico-legal purposes, research focusing on diabetes’ effects on quantification of chronic alcohol consumption has encountered some limitations, primarily because in scientific literature there is limited information and/or research focused on the production of endogenous ethanol and/or metabolization of exogenous alcohol in patients with diabetes. Furthermore, some authors [46] point out that in common practice, in particular for ethanol quantification in diabetic patients in cases of medico-legal/juridical interests, such as traffic violation, it has often happened that endocrinology specialists have been consulted who have provided very generic answers like “it cannot be excluded with certainty that the blood alcohol concentration depends on the disease of the suspected drinker”, without further clarification. Clearly, this data is not very useful in the forensic field, and it does not allow us to adequately distinguish between a drinker and a non-drinker in cases of diabetic patients. One of the problems related to the diabetic patient is the production of so-called “endogenous” ethanol. Endogenous ethanol is the result of spontaneous self-generation within the body itself; ethanol is formed from acetaldehyde within human body through various metabolic processes. This phenomenon is known as “physiological-blood-ethanol” [46]. This form of endogenous alcohol can derive from intra-intestinal fermentation of carbohydrates and from local flora’s action; this self-production of ethanol in intestine is also called “Auto-Brewery Syndrome”. Candida albicans, which can produce a maximum of 1 mg/h of ethanol per gram of intestinal content, for example, is generally the most responsible for the intestinal fermentation process. This amount of self-produced alcohol, as well as exogenous ethanol, is metabolized by the liver, which has an oxidation capacity of about 6–8 g of ethanol per hour in a young adult. It therefore emerges that the levels of ethanol in the blood of diabetic patients may undergo variations both due to the concomitant metabolic alteration typical of diabetic disease, but also in relation to blood glucose levels and/or the therapy to which the patient undergoes. 

In conclusion, these studies allow us to formulate the following considerations:

1. The quantification of EtG in hair is closely related to the amount of ethanol in the blood.
2. In diabetic patients, a statistically significant increase in endogenous ethanol production was found.
3. Ethanol production is affected by the presence of glucose in the blood.
4. The production of ethanol is also influenced by the simultaneous presence of infections, not uncommon in diabetic patients.
5. In diabetic patients there is a high risk of obtaining a false positive result.
6. No investigations have been found in scientific literature aimed at remodulating the cut-off in diabetic patients.
7. The data must be interpretated in any case, considering, contextually to EtG quantification, the quantification of glucose in blood, the presence of any infections and the type of germ responsible, and the antidiabetic therapy performed by the patient (e.g., insulin).

From the medico-legal point of view, ethanol’s quantification in diabetic patients must be critically evaluated and, above all, it requires further analytical studies, given the ever-increasing number of diabetic subjects, also in the attempt to be able to identify reference cut-offs for diabetic patients or even correction coefficients.

## 7. Summary and Conclusions

Correct quantification of ethanol, therefore, becomes indispensable from a forensic point of view [47,48]. Hair ethanol quantification is a useful and reliable laboratory investigation to detect alcohol intake or withdrawal. The accurate interpretation of data is essential to avoid false positives or false negative results. We have reviewed the literature and verified that some pathological conditions, such as hepatic, renal, and metabolic (such as diabetes) ones, have a decisive influence on the concentrations of ethanol in blood and consequently in the hair. Therefore, it is necessary to pay attention during the interpretation of the results and associate the analysis of the laboratory data with a medical history and careful clinical and documentary examination. In addition, we must consider that the common cut-offs used in the literature to discriminate between alcohol use, abuse and abstinence are not suitable for medico-legal purposes. At the current state, the studies in this regard are not exhaustive and an in-depth toxicological analysis is required to identify new cut-offs, calibrated in relation to the subjects’ different pathological conditions.

## Figures and Tables

**Table 1:** Results of investigations carried out by different authors; the laboratory techniques, sensitivity and specificity for each techique and respective cut-offs.

Authors, (Year) [Ref.]	Study Population	Amount of Hair (mg) or Sample Characteristics	Cut-Offs	Sensitivity-Specificity	Technique
Stewart et al. (2013) [39]	191	100	≥8 pg/mg		LC-MS/MS
			Any past 90-day drinking	58–99	
			Any past 90-day drinking among cirrhotic vs. non-cirrhotic	65–98 vs. 54–100	
			Average ≥ 28 g/day among cirrhotic vs. non-cirrhotic	100–94 vs. 82–80	
			≥30 pg/mg		
			Average ≥ 28 g/day	81–93	
Sterneck et al. (2014) [40]	88	n.s. (0.5 cm thick hair strand 3–6 cm long)	≥7 pg/mg		GC-MS
			Any past 180-day drinking	76–91	
			≥30 pg/mg		
			Any past 180-day drinking	85–97	
			Any past 90-day drinking	86–98	
Verbeek et al. (2018) [42]	144	n.s. (proximal 3-cm scalp hair strand)	≥7, <30 pg/mg		GC-MS/MS
			>0 g/day and <60 g/day in the past 90 days	67–66	
			≥30 pg/mg		
			>60 g/day in the past 90 days	100–97	
			≥50 pg/mg		
			>60 g/day in the past 90 days	100–100	

## Data Availability

Data sharing is not applicable; no new data were created or analyzed.
